# Multiclass Classification of Pigmented Skin Lesions Using a Multimodal Large Language Model

**DOI:** 10.7759/cureus.88711

**Published:** 2025-07-24

**Authors:** Kimi Iinuma, Kazuyasu Fujii, Chisa Nakashima, Kenichiro Kasai, Hiroyuki Irie, Hitonari Kanetomo, Shigeto Yanagihara, Sayuri Sato, Hisashi Uhara, Fumiaki Takeda, Atsushi Otsuka

**Affiliations:** 1 Dermatology, Kindai University Hospital, Osaka, JPN; 2 Plastic Surgery, Kasai Clinic for Plastic Surgery, Osaka, JPN; 3 Dermatology, Graduate School of Medicine, Kyoto University, Kyoto, JPN; 4 Dermatology, Kanetomo Dermatology Clinic, Osaka, JPN; 5 Dermatology, Sapporo Medical University School of Medicine, Sapporo, JPN; 6 Digital Monozukuri (Manufacturing) Education and Research Center, Hiroshima University, Hiroshima, JPN

**Keywords:** basal cell carcinoma (bcc), image classification, melanoma diagnosis, neoplasms, pigmented skin lesions

## Abstract

Background: Pigmented skin lesions span benign to malignant entities that often appear similar on standard clinical photographs, complicating accurate diagnosis without specialized imaging. Recently, multimodal large language models (MMLLMs) have attracted attention as image-based diagnostic aids and hold promise as decision-support tools in resource-limited settings where dermoscopy may be unavailable.

Objectives: This study aimed to determine whether a fine-tuned MMLLM can accurately classify eight common pigmented skin conditions using only clinical photographs, thereby providing a non-dermoscopic diagnostic support tool.

Methods: We fine-tuned InstructBLIP-flan-t5-xl (Salesforce, San Francisco, CA) using Hugging Face’s Seq2SeqTrainer (Hugging Face Inc., New York City, NY) on a curated dataset of 979 manually cropped regions of interest depicting one of eight lesion types (acquired dermal melanocytosis, basal cell carcinoma, ephelis, malignant melanoma, melasma, nevus, seborrheic keratosis, or solar lentigo). Images were split 80% for training and 20% for validation. During training, lesion labels were masked to encourage learning of visual-text correlations. Model performance was evaluated by macro-average sensitivity, specificity, F1 score, and area under the receiver operating characteristic area under the curve (ROC AUC) for each class.

Results: On the validation set, the model achieved a macro-average sensitivity of 86.0%, specificity of 98.2%, and F1 score of 0.86. ROC AUC exceeded 0.95 for six of eight classes. Malignant melanoma showed the highest performance (sensitivity 94%, ROC AUC 0.98), while nevus exhibited the lowest sensitivity (78%, ROC AUC 0.89).

Conclusions: Fine-tuned MMLLMs can accurately classify common pigmented skin lesions from clinical photographs alone, enabling rapid diagnostic support in environments lacking dermoscopy. Future work should expand dataset diversity, undertake multicenter validation, and assess real-world clinical utility to confirm broader applicability.

## Introduction

Accurate differentiation of pigmented skin lesions is of paramount importance for the early detection and selection of appropriate treatment for skin cancers, including malignant melanoma. Pigmented skin lesions encompass both benign and malignant entities, and their differential diagnosis can be extremely challenging [1,2]. Benign pigmented skin lesions are extremely prevalent, with approximately 11.6% of adults reported to have more than 50 acquired melanocytic nevi [3]. Conversely, malignant melanoma incidence has risen globally, with an estimated 325,000 new cases (age-standardized incidence rate ~3.2 per 100,000) diagnosed worldwide in 2020 [4]. While laser therapy is generally considered a treatment option for benign pigmented lesions, it may exacerbate certain conditions such as melasma. Moreover, misdiagnosing a malignant tumor as benign may delay timely surgical intervention. Therefore, achieving an accurate diagnosis is crucial, as it significantly impacts the patient's quality of life.

Traditionally, most machine-learning-based image classification models in dermatology have relied on dermoscopic images, achieving diagnostic performance comparable to that of experienced dermatologists [5,6]. However, obtaining dermoscopic images requires specialized equipment, limiting their use in routine clinical practice and resource-constrained environments [7,8].

Recently, multimodal large language models (MLLMs) have garnered attention and shown promise in medical image interpretation [7,9]. These models can integrate visual and textual information, enabling them to generate contextually rich responses to natural language queries based on image content. In particular, frameworks such as Flamingo and InstructBLIP have demonstrated strong task adaptation and few-shot learning capabilities in diagnostic contexts [7-10].

In this study, we focus on the underexplored application of MLLMs for differentiating pigmented lesions using only standard clinical photographs. We applied the InstructBLIP model to classify eight pigmented skin conditions: acquired dermal melanocytosis (ADM), basal cell carcinoma, ephelis (freckles), malignant melanoma, melasma, nevus, seborrheic keratosis, and solar lentigo. These eight categories were deliberately chosen to reflect a balance of clinical relevance, diagnostic complexity, and prevalence, ensuring that the classification task aligns closely with real-world dermatological challenges. By directly employing photographs captured in routine clinical settings, this approach suggests the feasibility of deploying MLLM-based decision-support tools in real-world dermatology.

## Materials and methods

Clinical images were captured under standardized lighting and framing conditions using a digital camera and stored as high‑resolution digital files. All clinical images were collected in compliance with institutional ethical guidelines. This study was approved by the Institutional Review Board of Kindai University (approval number: R06-074). Eight pigmented skin conditions were included: ADM, basal cell carcinoma, ephelis (freckles), malignant melanoma, melasma, nevus, seborrheic keratosis, and solar lentigo. The number of images per category was as follows: ADM (136), basal cell carcinoma (113), ephelis (92), malignant melanoma (100), melasma (167), nevus (91), seborrheic keratosis (123), and solar lentigo (157). Prior to training, regions of interest (ROIs) containing lesions were manually cropped from each image.

For multiclass classification, we constructed a custom dataset of image-text pairs. The JSON annotation file (instructblip_data_noimage_fixed.json) (Labelbox Inc., San Francisco, CA) contained image file paths, user prompts, and assistant responses structured in a question-answer format related to skin diseases, yielding 979 usable samples. These eight categories were selected based on their clinical relevance and distinct visual and pathological features. The dataset was split into training (80%) and validation (20%) sets using a fixed random seed, ensuring no overlap of lesions or patients between subsets. To preserve class balance across subsets, stratified sampling was performed based on disease category during the split. Dynamic data augmentation (random horizontal and vertical flips and rotations) was applied during training; no augmentation was performed during dataset construction.

We adopted InstructBLIP (Salesforce, San Francisco, CA/instructblip-flan-t5-xl), a multimodal model comprising a vision encoder and a T5-based decoder, to handle combined image and text input. Tokenization and image preprocessing were performed using the InstructBlipProcessor (Salesforce). All images were resized to 224 × 224 pixels. Text prompts were formatted by appending “USER: {clinical image description}” followed by “ASSISTANT:”.

Training was conducted with HuggingFace Transformers’ Seq2SeqTrainer (Hugging Face Inc., New York City, NY) using a learning rate of 1 × 10⁻⁶, three epochs, and a batch size of 1. Mixed precision (fp16) was disabled for compatibility with Apple Silicon (Apple Inc., Cupertino, CA). Label padding tokens were set to -100 to exclude them from loss computation.

Model performance was monitored at each epoch on the validation set by computing macro‑averaged precision, recall, F1 score, sensitivity, specificity, receiver operating characteristic area under the curve (ROC AUC), and PR AUC. After training, trainer.predict() generated detailed predictions on both the validation and a held‑out test set comprising 20% of images per class from distinct patients (ADM: 34, basal cell carcinoma: 28, ephelis: 23, malignant melanoma: 25, melasma: 41, nevus: 23, seborrheic keratosis: 30, solar lentigo: 39). ROC and precision recall (PR) curves aggregating all classes were plotted in single figures, and the confusion matrix was visualized as a heatmap to assess misclassification patterns. Per-class metrics (PR, specificity, and F1 score) were saved to CSV files, and macro-average values were reported as summary performance metrics.

All code was implemented in Python using PyTorch (Facebook AI Research (FAIR), a part of Meta Platforms, Inc., Menlo Park, CA) and HuggingFace Transformers for model training and inference, scikit‑learn for metrics calculations, and Matplotlib (Matplotlib Organization) for visualization.

## Results

**Figure 1:**
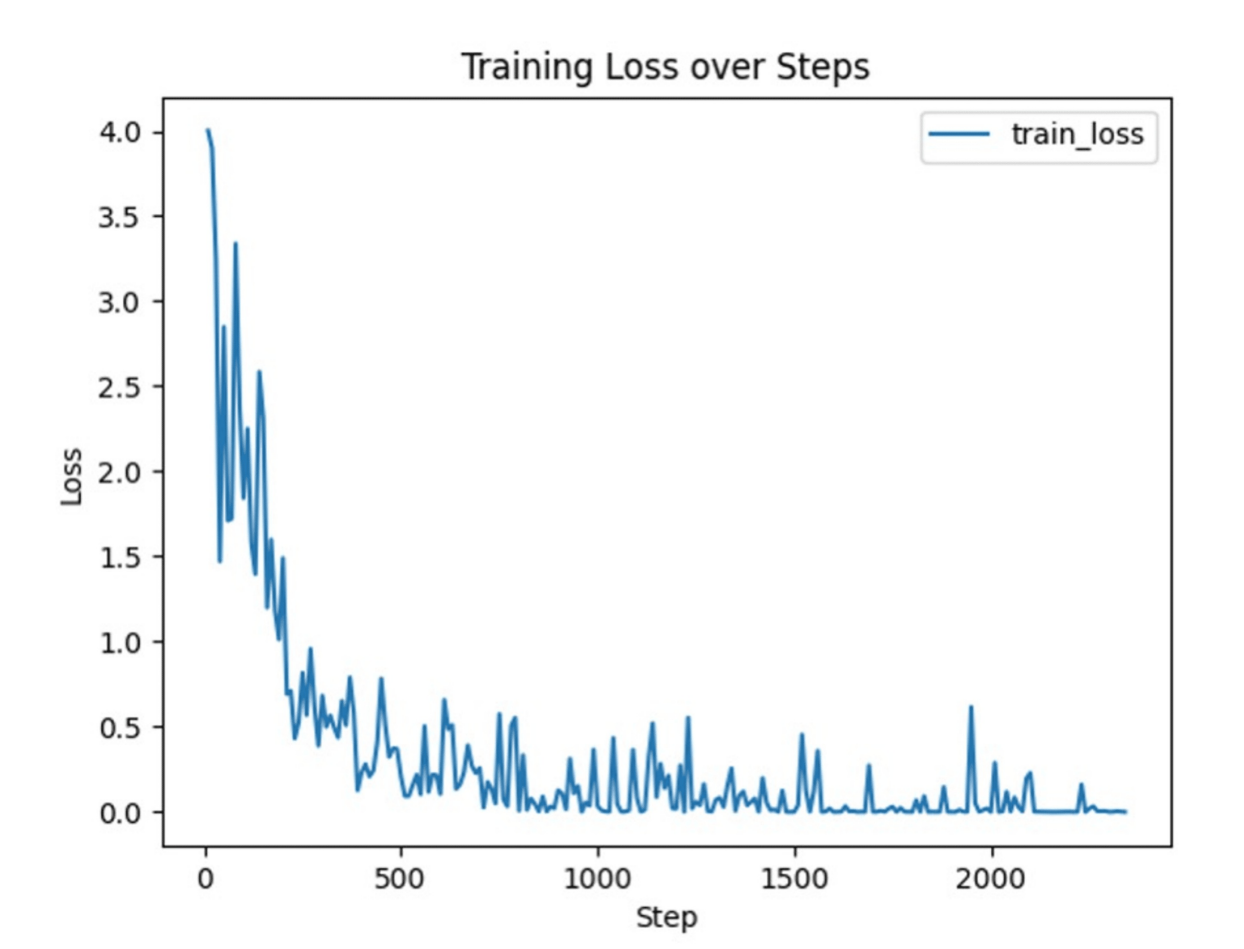
Training loss curve The graph illustrates the change in training loss over the course of model training. A steady decrease in loss indicates that the model successfully learned from the data, with no signs of divergence or instability during training.

As shown in Figure 1, the training loss exhibited a steady and smooth decrease across the three training epochs, reaching near-0 values by the end of the final epoch. This trend indicated stable model convergence and effective learning of relevant features from clinical images without signs of overfitting. The model demonstrated generalizability, as confirmed by the high validation performance metrics described below.

On the validation dataset, the model achieved a macro-averaged precision of 90.6%, recall of 90.3%, specificity of 98.5%, and F1-score of 0.903. On the independent test set, performance remained robust, with a precision of 86.1%, a recall of 86.0%, a specificity of 98.2%, and an F1 score of 0.860, suggesting minimal performance degradation across datasets. These metrics are summarized in Tables 1, 2, which also provide per-class performance. Notably, melasma, ephelis, and solar lentigo consistently exhibited high scores across all metrics, whereas nevus and seborrheic keratosis showed slightly reduced recall values on the test set, reflecting the inherent diagnostic challenge of visually similar or less distinct lesion types.

**Table 1:** Per-class classification metrics on the validation set Precision, recall, specificity, and F1 score are reported for each of the eight lesion categories based on predictions from the validation dataset. Melasma, ephelis, and nevus demonstrated particularly high scores, reflecting strong performance on clinically distinguishable lesions.

Variables	Precision	Recall	Specificity	F1 score
Acquired dermal melanocytosis (ADM)	0.806	0.926	0.964	0.862
Basal cell carcinoma	0.846	0.88	0.977	0.863
Ephelis	1	0.944	1	0.971
Malignant melanoma	0.889	0.889	0.989	0.889
Melasma	0.968	1	0.994	0.984
Nevus	1	0.882	1	0.938
Seborrheic keratosis	0.826	0.905	0.977	0.864
Solar lentigo	0.914	0.8	0.981	0.851

**Table 2:** Per-class classification metrics on the test set Classification metrics for each lesion type were evaluated on the independent test dataset. While overall performance remains high, nevus and seborrheic keratosis show slightly reduced recall values, indicating challenges in distinguishing visually similar lesions.

Variables	Precision	Recall	Specificity	F1 score
Acquired dermal melanocytosis (ADM)	0.971	0.971	0.995	0.971
Basal cell carcinoma	0.857	0.857	0.981	0.857
Ephelis	0.783	0.783	0.977	0.783
Malignant melanoma	0.88	0.917	0.986	0.898
Melasma	0.878	0.878	0.975	0.878
Nevus	0.762	0.696	0.977	0.727
Seborrheic keratosis	0.781	0.833	0.967	0.806
Solar lentigo	0.974	0.949	0.995	0.961

**Figure 2:**
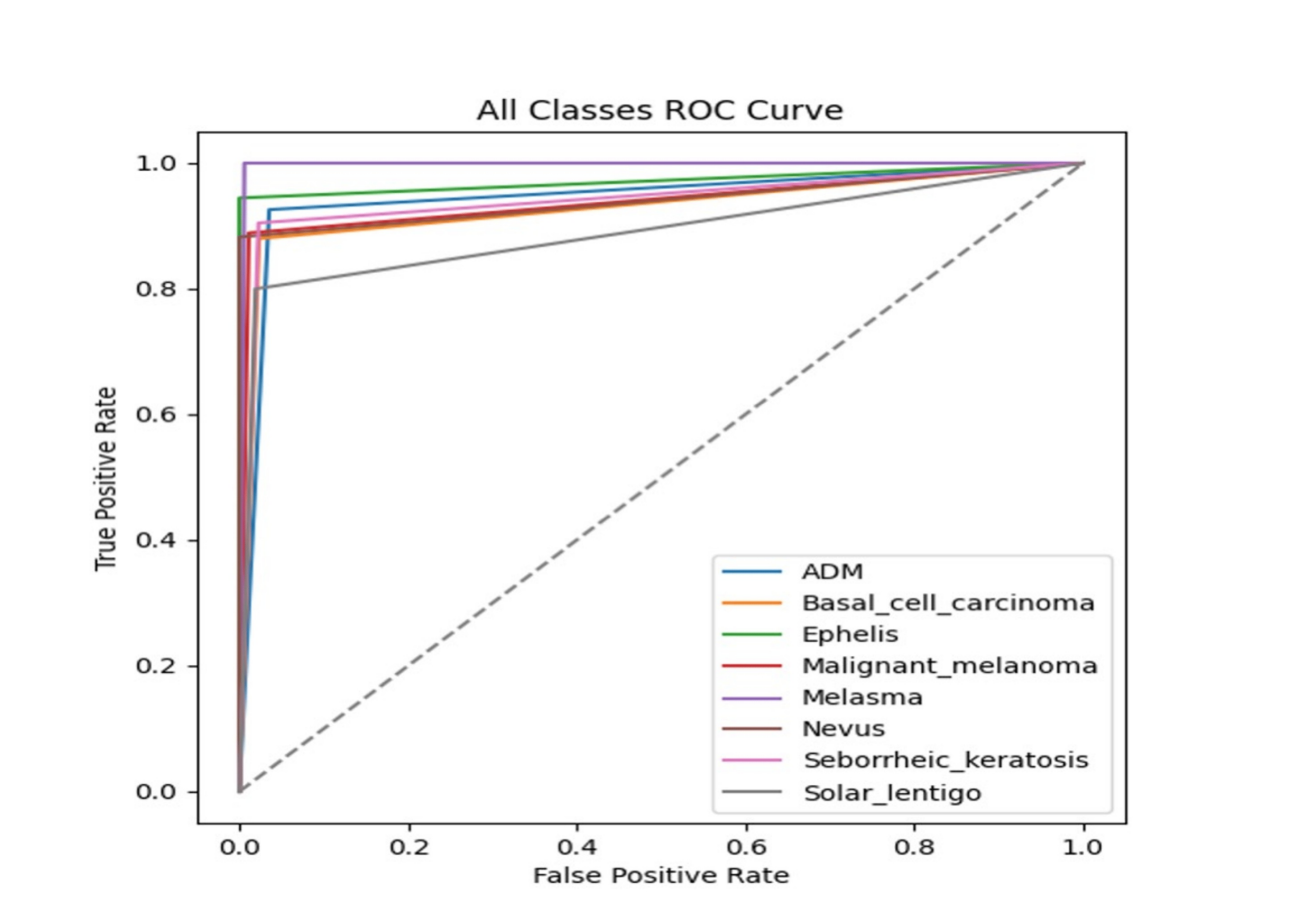
Receiver operating characteristic (ROC) curves for all lesion categories on the validation set ROC curves for the eight lesion categories on the validation dataset. Most curves reach close to the top-left corner, indicating high sensitivity and specificity. The macro-average ROC area under the curve (AUC) is 0.944, suggesting strong overall discriminative performance. ADM: acquired dermal melanocytosis

The ROC curves for all eight lesion categories were presented in Figure 2. The model demonstrated macro-average ROC AUCs of 0.944 and 0.921 for the validation and test sets, respectively, exceeding the commonly accepted threshold for high discriminative ability. Most curves approached the top-left corner, indicating high true-positive rates and low false-positive rates across categories. Particularly, the ROC curves for melasma, malignant melanoma, and ephelis were steep and close to optimal, reflecting confident classification.

**Figure 3:**
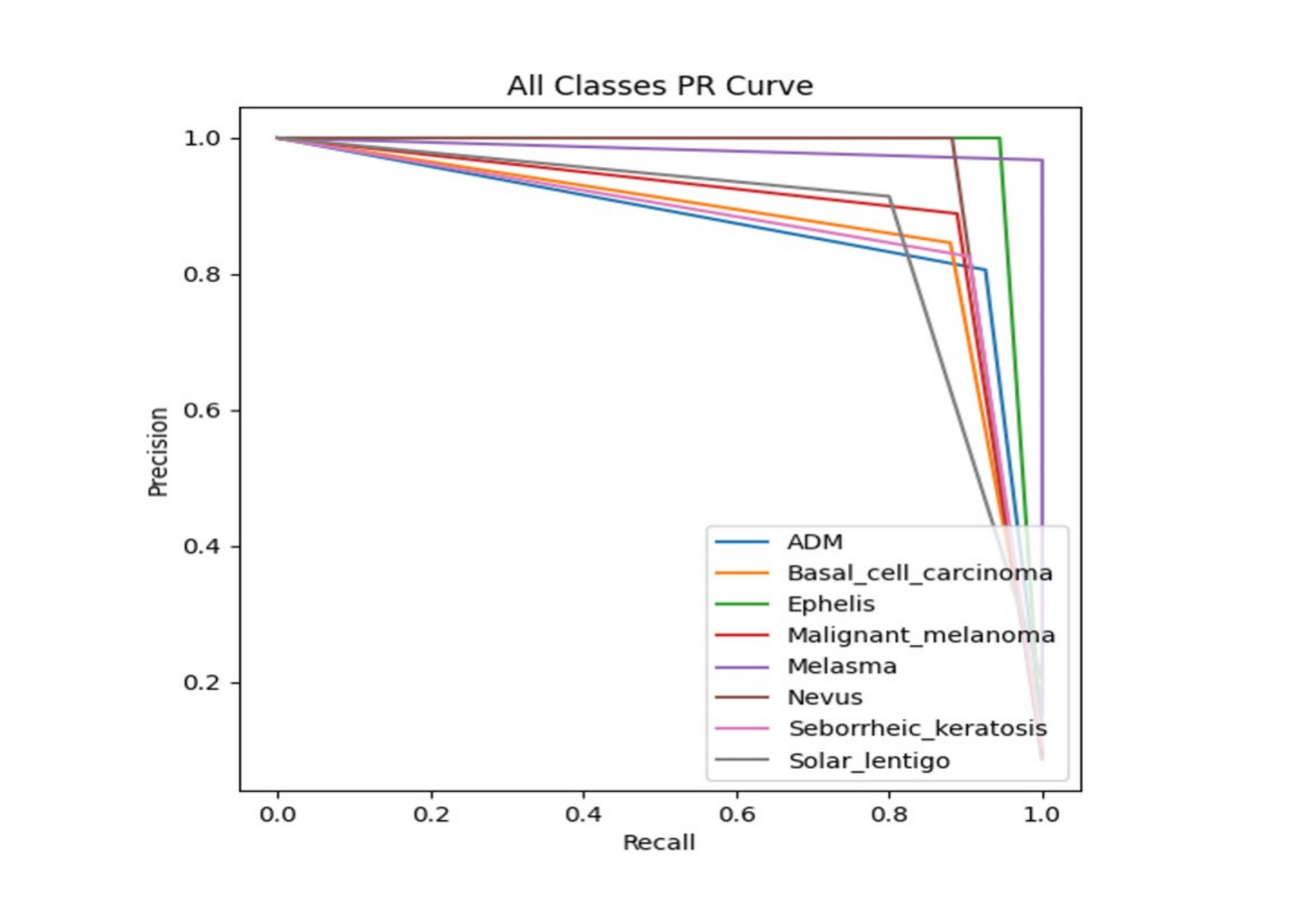
Precision–Recall (PR) Curves for Eight Lesion Categories on the Validation Set PR curves showing model performance under varying recall thresholds. The macro-average PR area under the curve (AUC) exceeds 0.9, and precision remains above 80% even at high recall levels, indicating robustness in sensitivity-prioritized diagnostic scenarios such as melanoma screening. ADM: acquired dermal melanocytosis

To further assess model performance in high-sensitivity contexts, Figure 3 shows the PR curves for all lesion categories. Across both datasets, precision remained above 80% at recall levels greater than 0.8, indicating that the model maintains reliable precision even when operating in a sensitivity-prioritized diagnostic setting. The macro-average PR AUC exceeded 0.9 on both datasets. These findings were particularly relevant in clinical applications such as melanoma screening, where high sensitivity is essential to minimize the risk of missed malignancies.

**Figure 4:**
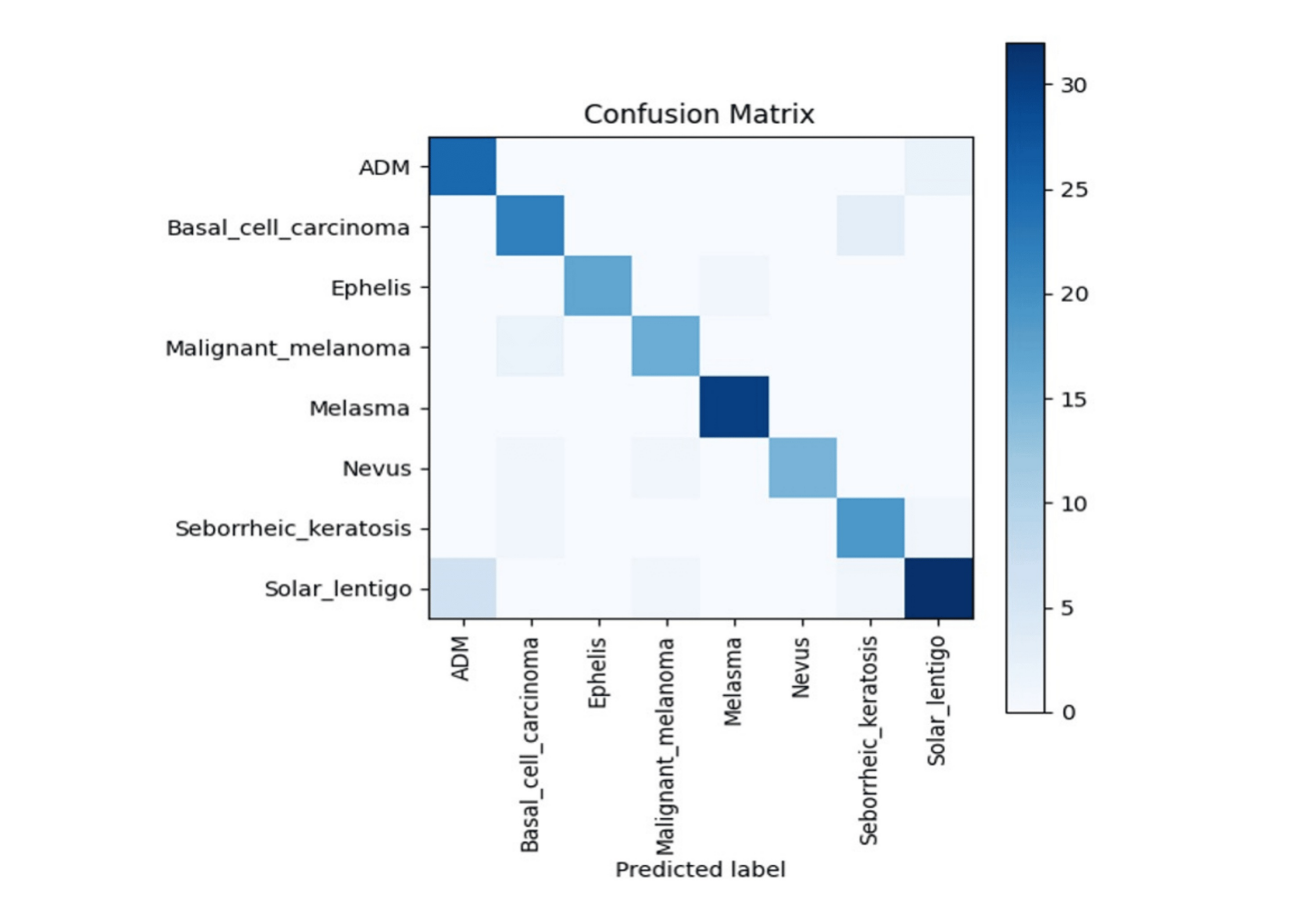
Confusion matrix for multiclass classification on the test dataset The confusion matrix illustrates model predictions versus true labels. Most misclassifications occurred between acquired dermal melanocytosis (ADM) and solar lentigo, and between basal cell carcinoma and seborrheic keratosis, reflecting similar clinical appearances. Malignant melanoma has relatively few errors, showing distinctiveness in its visual patterns.

Misclassification patterns were further explored using the confusion matrix shown in Figure 4 (test set). The most notable confusion occurred between ADM and solar lentigo, as well as between basal cell carcinoma and seborrheic keratosis, likely reflecting similar coloration and textural features in clinical photographs. In contrast, malignant melanoma exhibited relatively fewer misclassifications, suggesting that the model effectively captured its distinct visual cues. These patterns aligned with known diagnostic challenges even among human clinicians.

As detailed in Tables 1, 2, lesion types with well-defined borders and uniform pigmentation, such as ephelis, melasma, and solar lentigo, achieved F1-scores above 0.90, reflecting consistent recognition. Conversely, nevus and seborrheic keratosis, which exhibited greater intraclass heterogeneity and overlapping features with other categories, showed moderately lower recall (0.696 and 0.833, respectively). This observation highlighted the potential benefit of incorporating dermoscopic data or clinical metadata to resolve ambiguities.

## Discussion

This study successfully applied the multimodal large language model InstructBLIP to perform multiclass classification of pigmented skin lesions using only standard clinical photographs, achieving high overall accuracy, sensitivity, and specificity. Notably, benign lesions that are often visually subtle, such as melasma and ADM, yielded high F1 scores, suggesting that InstructBLIP effectively learned fine-grained differences in color and pattern.

Previous research has predominantly relied on dermoscopic images [5,11], leveraging their detailed image information to attain high classification accuracy. Many dermoscopy-based studies report sensitivities for malignant melanoma exceeding 90%, and in our clinical-photograph-based approach, the sensitivity for malignant melanoma reached 0.917, which is comparable to that of dermoscopy-trained classifiers. This result implies that MLLMs can integrate natural language and visual inputs to replicate aspects of clinicians’ contextual interpretation of skin lesions.

Furthermore, when compared to Pacheco et al., who achieved a balanced accuracy of 0.718 and an AUC of 0.948 by combining smartphone‑acquired clinical photographs with patient information, our model demonstrated superior classification performance using only clinical photographs [12].

However, sensitivity was somewhat lower for categories such as nevus and seborrheic keratosis, indicating that discriminating between benign lesions with similar coloration or indistinct borders remains challenging when relying solely on clinical photographs. In such cases, incorporating dermoscopic imaging may further enhance diagnostic performance. As reflected in the confusion matrix, misclassifications between ADM and solar lentigo or between basal cell carcinoma and seborrheic keratosis occurred in lesion pairs with comparable color depth and ambiguous boundaries, suggesting that the critical surface details necessary for differentiation may have been obscured in 224 × 224-pixel crops.

Although several deep-learning studies using only clinical photographs have reported high AUCs (e.g., 0.96 for malignant melanoma and basal cell carcinoma) and diagnostic accuracy comparable to dermatologists [13], our study is the first to apply an MLLM across both benign and malignant pigmented lesion categories. Our results demonstrate that high-accuracy multiclass classification of pigmented skin lesions from clinical photographs alone is feasible and may extend the utility of artificial intelligence (AI)-based diagnostic support beyond the dermoscopy paradigm.

A notable advantage of our approach is its low barrier to clinical adoption, as it requires no specialized equipment beyond standard digital cameras or smartphones. This accessibility supports deployment in resource-limited settings and teledermatology, where patients can submit smartphone-captured images for remote evaluation, thereby improving access to dermatological care. Recent advances in MLLM research also explore the integration of image analysis with patient clinical data, enabling interactive prompting to interrogate diagnostic rationale and support human clinical reasoning. Han et al. demonstrated that MLLMs interpret medical images with contextual understanding beyond simple pattern recognition [7], consistent with our findings. Looking forward, MLLM-based systems could be developed as chatbot-like diagnostic assistants capable of answering patient questions and providing explanations of their decision-making process.

In addition to multimodal input from clinical photographs and language, future models may benefit from integrating structured clinical metadata such as patient age, lesion duration, or anatomical site, which are routinely available in electronic health records. Semi-supervised learning strategies could also be adopted to leverage larger volumes of unlabeled images, further enhancing model generalizability.

Moreover, ethical considerations must be addressed prior to the clinical deployment of AI systems. These include the risk of misdiagnosis, the potential for algorithmic bias across demographic groups, and the necessity of clinician oversight to ensure safe and responsible use in clinical settings.

To further enhance generalizability and real-world applicability, future studies should include multi-center datasets encompassing diverse skin phototypes and clinical settings. External validation using independent cohorts will be prioritized as the next step.

Limitations

The dataset of 979 images from a single institution is subject to demographic and skin phototype bias; insufficient representation of diverse skin tones and racial backgrounds may reduce model fairness and accuracy when applied to broader patient populations. Many prior dermatology AI studies have been criticized for training predominantly on Caucasian skin images, with reported declines in performance on darker skin tones [14-16].

Manual ROI cropping was required for model input. For clinical implementation, an automated lesion detection pipeline must be developed; the model’s performance on full-frame images containing multiple lesions or varied backgrounds without prior ROI extraction remains untested. Additionally, variability in image quality and resolution across devices and settings poses challenges for standardization and consistent diagnostic performance.

## Conclusions

This study demonstrates the feasibility of high‑accuracy multiclass classification of pigmented skin lesions using an MMLLM applied solely to clinical photographs, underscoring its practical applicability in routine dermatology and potential for telemedicine deployment.

Future directions include multimodal integration with dermoscopic imaging and patient history data, prospective validation on real-world clinical datasets, and evaluation of the model’s usability by non-specialist clinicians. Moreover, the model’s transfer-learning capability enables effective fine-tuning on relatively small datasets, suggesting high adaptability to rare diseases and region-specific lesion types.
